# On the effect of changing handgrip position on joint specific power and cycling kinematics in recreational and professional cyclists

**DOI:** 10.1371/journal.pone.0237768

**Published:** 2020-08-19

**Authors:** Knut Skovereng, Lorents Ola Aasvold, Gertjan Ettema

**Affiliations:** Department of Neuromedicine and Movement Science, Centre for Elite Sports Research, Norwegian University of Science and Technology, Trondheim, Norway; University of Bourgogne France Comté, FRANCE

## Abstract

**Introduction:**

In cycling, the utilization of the drops position (i.e. the lowest handlebar position relative to the ground) allows for reduced frontal area, likely improved aerodynamics and thus performance compared to the tops (i.e. the position producing the most upright trunk). The reduced trunk angle during seated submaximal cycling has been shown to influence cardiorespiratory factors but the effects on pedalling forces and joint specific power are unclear. The purpose of this study was to investigate the effect of changing handgrip position on joint specific power and cycling kinematics at different external work rates in recreational and professional cyclists.

**Method:**

Nine professional and nine recreational cyclists performed cycling bouts using three different handgrip positions and three external work rates (i.e. 100W, 200W and external work rate corresponding to the lactate threshold (WR_lt_)). Joint specific power was calculated from kinematic measurements and pedal forces using 2D inverse dynamics.

**Results:**

We found increased hip joint power, decreased knee joint power and increased peak crank torque for the professional cyclist compared to the recreational cyclists, but only at WR_lt_ where the professional cyclists were working at a higher external work rate. There was no main effect of changing handgrip position on any joint, but there was a small interaction effect of external work rate and handgrip position on hip joint power contribution (Generalized eta squared (η_g_^2^) = 0.012). At 100W, changing handgrip position from the tops to the drops decreased the hip joint contribution (-2.0 ± 3.9 percentage points (pct)) and at the WR_lt_, changing handgrip position increased the hip joint power (1.6 ± 3.1 pct). There was a small effect of handgrip position with the drops leading to increased peak crank torque (η_g_^2^ = 0.02), increased mean dorsiflexion (η_g_^2^ = 0.05) and increased hip flexion (η_g_^2^ = 0.31) compared to the tops.

**Discussion:**

The present study demonstrates that there is no main effect of changing handgrip position on joint power. Although there seems to be a small effect on hip joint power when comparing across large ranges in external work rate, any potential negative performance effect would be outweighed by the aerodynamic benefit of the drops position.

## 1. Introduction

In road cycling, high speeds makes air drag a primary source of external resistance and utilizing an aerodynamically efficient position is often essential to achieve an optimal performance. On the handlebar of a standard road bike, the drops position is the lowest in relation to the ground. Using the drops compared to the tops (i.e. the handgrip position furthest from the ground and towards the back of the bike) and hoods (i.e. same height as tops, but further towards the front of the bike) will reduce the inclination of the trunk which in turn will reduce the frontal area of the cyclists [1] and thus the air resistance [2].

Ashe et al. showed that for untrained subjects, VO_2max_ and peak power output is reduced and oxygen cost of submaximal cycling is increased when using a horizontal trunk and potentially more aerodynamically efficient position compared to a vertical trunk position [3]. In contrast, Grappe et al. showed no physiological effects of using the drops compared to an hoods position in competitive cyclists [4] which indicates that there may be an effect of athlete level where the competitive cyclists adapt and are potentially less affected by a reduced trunk angle, at least for the physiological response.

Potentially underlying the mentioned metabolic effects, and also influenced by change in handgrip position, is pedalling technique. Frequently, the investigation of pedalling technique includes muscle activation, movement kinematics and pedal forces. An expected effect of changing handgrip position from the tops to the drops is kinematic changes such as increased anterior pelvic tilt [5]. Using a time trial position has been shown to elicit greater gluteus maximus and vastus lateralis muscle activity in trained cyclists [6] as well as increased co-activation in novice cyclists [7] compared to the tops position. Fintelman et al. [8] demonstrated that utilizing a horizontal trunk position influenced pedal forces and led to increased and delayed peak crank torque during the pedalling cycle compared to a 16 degree inclined trunk in trained time trial cyclists. Chapman et al. [7] showed an effect of changing from the hoods to the drops position on the muscle activity of novice cyclists but not trained cyclists. Taken together, the literature indicates that there is an effect of upper body position on variables related to technique and coordination and that there seems to be differences between cyclists of different levels.

Using a combination of pedal forces and kinematic measurements, inverse dynamics can be used to calculate joint specific power production, more specifically in seated cycling, the amount of power generated in the hip, knee and ankle joints. Investigating the hip, knee and ankle joint contribution may provide additional insight into how pedalling technique is affected by a change in body orientation. To the best of our knowledge, the effect of different handgrip positions on joint specific power during seated submaximal cycling has not been previously investigated. However, inverse dynamics has previously been used to study the effect of multiple cycling related factors such as external work rate [9–11] and cadence [12–14]. Bini et al. [15] investigated the joint contribution to leg work during sprint cycling at different handlebar positions and showed increased hip work and decreased knee work at the lowest handlebar positions. Sprint cycling is characterized by very high external work rates, but for the effects related to intensities used in daily training or longer competitions, the effects of handgrip positions during submaximal intensities are perhaps of greater interest.

Therefore, the purpose of this study was to investigate the effect of cycling handgrip position at different power outputs on joint specific power in cyclists ranging from recreational to elite level. Additionally, we investigated the effects of changing handgrip position on kinematic (e.g. joint angles) and kinetic variables that could help explain the findings on joint specific power.

## 2. Methods

### 2.1 Subjects

Nine professional male cyclists, recruited from a continental level team and nine recreational male cyclists, recruited from local clubs participated in the study (Table 1). The study was registered and approved by Norwegian Social Science Data Services (Pnr: 50084). All participants signed a written consent form after receiving information about the study. The participants were informed explicitly that they could withdraw at any time without stating a reason. The study was conducted in accordance with the general principles outlined in the Declaration of Helsinki.

**Table 1:** Subject characteristics.

	Professional			Recreational			*p*	*g*_*s*_
Age (years)	22	±	1.5	40	±	9.6	<0.01	2.48
Body mass (kg)	73.4	±	7.8	85.8	±	9.2	<0.01	1.38
Height (cm)	183	±	5	184	±	6	0.58	0.25
Peak heart rate (bpm)	201	±	5	191	±	5	<0.01	2.02
WR_lt_ (W)	315	±	23	250	±	19	<0.01	2.98
WR_lt_ (W/kg)	4.3	±	0.5	2.8	±	0.4	<0.01	3.28

Age (years), Body mass (kg), Height (cm), Peak heart rate (beats per minute), external work rate corresponding to a blood lactate of 4mmol/l presented as absolute power (W) and relative to body mass (W/kg). g_s_ = Hedges g_s_.

### 2.2 Experimental protocol

All participants visited the laboratory on two occasions. The first day consisted of anthropometric measurements in addition to familiarize the participants with the laboratory setting through an incremental exercise test, consisting of multiple 5-minute stages with increasing intensity (25W per stage) and a freely chosen cadence was used, in order to determine lactate threshold (LT) corresponding to an intensity eliciting a blood lactate of 4 mmol·L^-1^ (WR_lt_). The incremental test was ended when blood lactate exceeded 4 mmol·L^-1^.

The second day consisted of a warm up (~15 minutes at a self-selected external work rate characterized as low intensity), followed cycling at three different intensities (i.e. 100W, 200W and WR_lt_) using three different handgrip positions (i.e. the tops, the hoods and the drops) at a freely chosen cadence. The participants were given approximately 30 to 60 seconds during each condition to adjust to the external work rate and stabilize their pedalling prior to kinematic- and pedal force-measurements. Kinematic data and pedal forces were measured for 30 seconds starting after a steady state external work rate was achieved and the participants were unaware of the exact periods for measurements. If the total time for reaching a steady state of pedalling and data collection exceeded 120 seconds, the measurement was redone in order to minimize the influence of fatigue during each trial. A freely chosen cadence was used during all cycling and the resistance was automatically adjusted to achieve the desired external work rate.

### 2.3 Instrumentation

All cycling was done using the participants’ personal road bikes with drop bars and an electronically braked trainer ergometer (Computrainer Lab_TM_, Race Mate, Seattle, WA). The tire pressure was manually pumped to a standardized 100 psi and the room temperature was held at approximately 19 degrees Celsius. The ergometer was calibrated according to the manufacturers’ specifications and controlled using specialized software (PerfPRO Studio, Hartware Technologies, Rockford, MI).

Pedal forces was measured using custom made force pedals equipped with two force cells (Revere Model 9363, capacity 250 kg per cell, the Netherlands) with 2x2 degrees of freedom and a sampling frequency of 100Hz. The pedals were calibrated by hanging weights from the pedal axis (i.e. 2.5–15 kg) with the pedal fastened in two orientations, thus applying normal and shear forces and measured cross-talk was proportional and remained <3%. A measurement of zero load was done prior to each data collection.

An 8-camera 3D motion capture system was used for kinematic analysis (Oqus 400, Qualisys, Sweden). Reflective markers placed on the neck (cervical spine), pelvis (iliac crest), hip (greater trochanter), knee (lateral epicondyle), ankle (lateral malleolus) and on the front and back of custom-made extensions placed symmetrically on the pedal spindle (see [16] for a picture). Data from the instrumented pedals and motion capture system was recorded simultaneously using Qualisys Track Manager (Qualisys, Sweden) at a sampling rate of 100 Hz.

### 2.4 Data analysis

**Fig 1:**
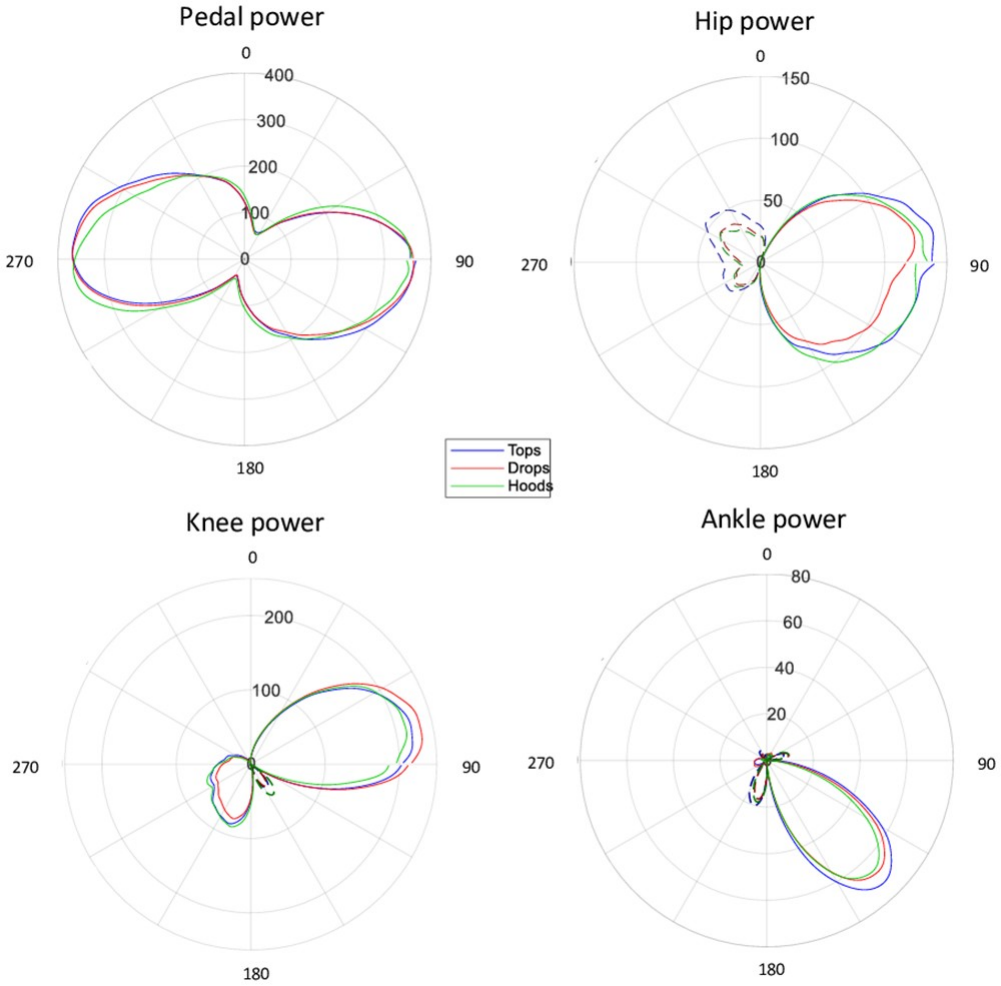
Polar plots for pedal power (W, both legs combined) and hip, knee and ankle joint power (W, one leg only) presented as a function of crank angle for three handgrip positions at 200W for a representative subject. Pedal cycle is clockwise. Labels inside the plots indicate y-levels.

A low pass filter was applied to the data before further analysis (10 Hz, 8th order, zero lag Butterworth). The complete description of the analysis has been published earlier [16], but we describe it here in brief. Power was calculated as the product of effective (perpendicular) crank force and crank angular velocity. Continuous crank angular velocity was calculated from crank angles using a 5-point differentiating filter. The average crank cycle (for all variables) was calculated by expressing all data of all cycles completed during the 30 second recording against crank angle and performing an averaging interpolation of these data, reducing the data set to 360 samples (i.e., 1 sample per degree crank angle). The markers placed on the pedal spindle were used to calculate pedal position, pedal orientation and crank angle. Pedal normal and shear forces were transformed to crank normal and shear forces by rotation of the coordination system from pedal to crank using the angle between pedal and crank as calculated from the kinematic data. Joint powers for the hip, knee and ankle joints were calculated using two-dimensional inverse dynamics for a linked system of rigid segments from pedal forces, body segment movements [16–18] and corresponding inertial estimates [19]. Thigh, leg and foot mass was calculated as 10.3, 4.3 and 1.5% of body mass respectively and segment centre of mass was calculated as 43, 43 and 27% of thigh, leg and foot length respectively measured from the cranial end (Van Soest et al. [19]). Relative mean joint power was calculated as the percentage contribution of each joint to the sum of hip, knee and ankle joints when averaged over the entire pedal cycle. Joint work was calculated as the amount of work done at each joint in joules in one complete pedal revolution. Fig 1 shows an example of the polar plots of raw data presented as a function of crank angle for the three handgrip positions at 200 W.

Mean joint angle and range of motion was calculated from reflective markers places on abovementioned anatomical positions. Because joint angles calculated from markers placed on the skin or tight fitting clothing above palpated anatomical position, it should be mentioned that there may be deviation between true joint angles and the measured joint angles due to sliding of the skin during movement and variation resulting from palpation. Ankle angle was calculated from the markers of the knee, ankle and pedal with the marker at the lateral malleolus as joint centre. Hip angle was calculated from the markers of the pelvic, hip and knee with the marker at the greater trochanter as joint centre.

### 2.5 Statistics

All descriptive data are presented as mean ± standard deviation. In order to evaluate the effect of handgrip position, external work rate and performance level on joint specific power and work, cadence, torque and kinematics the main analysis was done using a mixed design three-way repeated measures ANOVA. Handgrip position and external work rate was evaluated as within subject factors and athlete level was evaluated as between subject factor. If significant main and interaction effects were found, pairwise comparisons using T-test with Bonferroni correction was used to evaluate specific effects between external work rates, handgrip positions and athlete level. If the assumption of sphericity was violated, p-values were adjusted using the Greenhouse-Geisser correction. Strengths of association in the main analysis were quantified by generalized eta squared (η_g_^2^) and categorized as trivial/none (<0.0099), small 0.0099–0.0588), moderate (0.0588–0.1379), and large effect (>0.1379). When comparing means, effect size was calculated as Hedges g_s_ (g_s_) due to the limited number of participants in each group and assessed as of small (0.1–0.3), moderate (0.3–0.5), and large effect (>0.5) [20]. All data analysis and statistical analysis was conducted using Excel for Windows, SPSS 25.0 (SPSS, Chicago, USA) for WINDOWS, Matlab R2017b (MathWorks Inc. Natic, USA). Statistical significance was accepted at p < 0.05.

## 3. Results

### 3.1 Cycling conditions

The WR_lt_ was, as expected, higher (i.e. 266 ± 21 W vs 331 ± 25 W) for the professional group (p < 0.01, *g*_*s*_ = *2*.*68*). For cadence, there was a moderate main effect of external work rate (p<0.01, η_g_^2^ = 0.14) with WR_lt_ leading to a 6.4 RPM lower cadence compared to 100W and significant but trivial main effect of handgrip position (p<0.01, η_g_^2^ = 0.008) with the drops leading to 1.6RPM higher cadence compared to the tops.

### 3.2 Joint specific power

**Fig 2:**
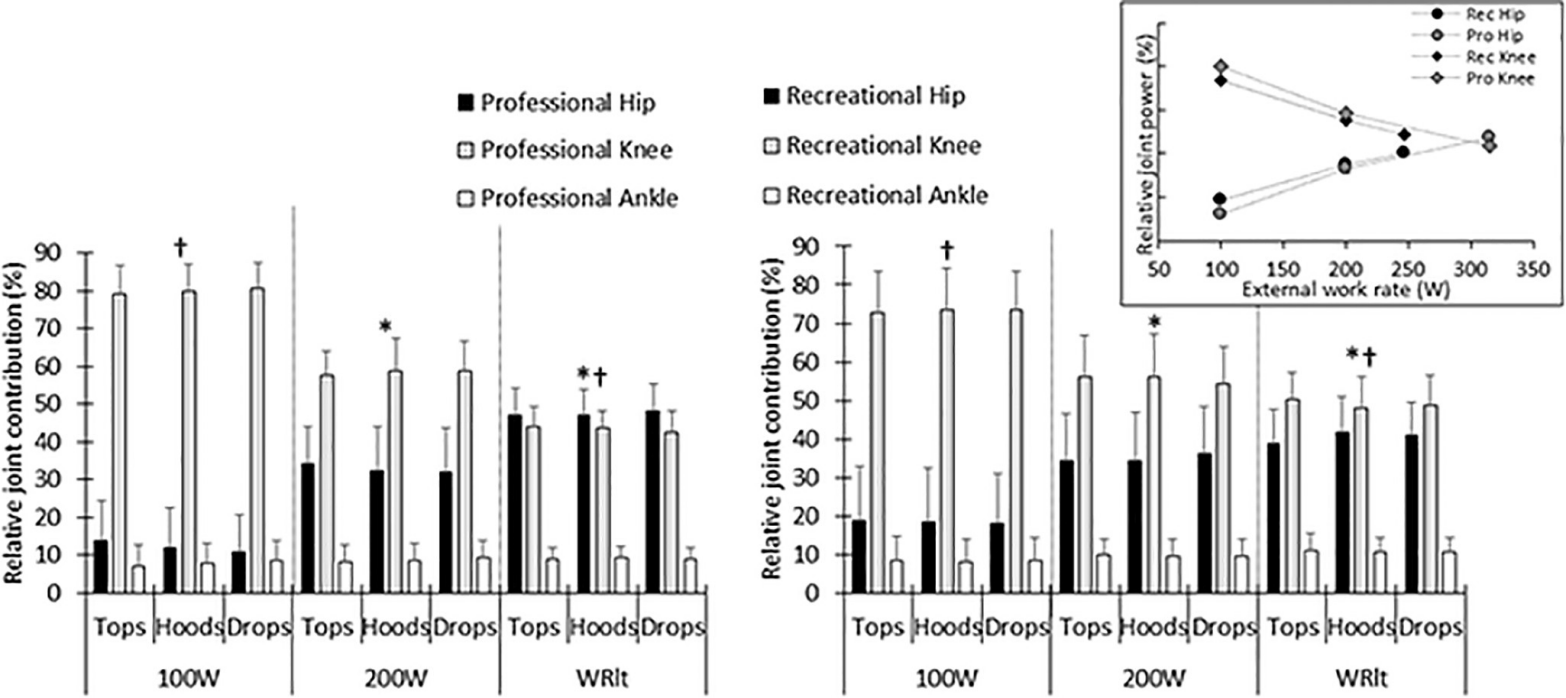
Joint specific power for the hip (black bars), knee (grey bars) and ankle (white bars) joints in the tops, hoods and drops positions for the professional (left panel) and recreational (right panel) cyclists. *indicates a significant difference from previous external work rate, # indicates a significant interaction between external work rate and performance level and † indicates a significant interaction effect between external work rate and handgrip position. Inset plots the hip (circles) and knee (diamonds) joints for both groups as a function of absolute external work rate.

There was a large main effect of increasing external work rate on joint specific power (Fig 2) leading to decreased mean relative knee joint power (p < 0.01, η_g_^2^ = 0.87) and increased mean relative hip joint power (p < 0.01, η_g_^2^ = 0.80) but there was no effect on ankle power (p = 0.13, η_g_^2^ = 0.10). There was no main effect of handgrip position (all p > 0.27 and all η_g_^2^ < 0.01) or athlete level (all p > 0.64 and all η_g_^2^ < 0.01) on hip, knee or ankle relative joint power.

There was a large interaction effect of athlete level and external work rate for hip joint (p < 0.01, η_g_^2^ = 0.21) and knee joint power (p < 0.01, η_g_^2^ = 0.18). Pairwise comparisons showed that when comparing 200W to WR_lt_, the professional cyclists had a greater increase in relative joint power in the hip (mean difference: 9.11 percentage points (pct), p < 0.01, g_s_ = 1.36) and a greater reduction in the relative joint power in the knee (mean difference: 8.43 pct, p < 0.01, g_s_ = 1.43) compared to the recreational cyclists.

There was also a small interaction effect of handgrip position and external work rate for the hip joint (p < 0.05, η_g_^2^ = 0.012). Pairwise comparisons showed that a change in handgrip from the tops to the drops reduced hip joint contribution at 100W (-2.0 ± 3.9 pct) and increased it at WRlt (1.6 ± 3.1 pct) (p < 0.05, g_s_ = 0.97).

There was no 3-way interaction between handgrip, athlete level and external work rate for any of the three joints (all p > 0.30 and all η_g_^2^ < 0.004).

### 3.3 Joint specific work

Since there was an effect of external work rate on cadence, we present absolute joint specific work (Table 2). There was a large main effect of external work rate (p<0.01, η_g_^2^ = 0.91) leading to increased joint work for all joints (all p<0.01, all η_g_^2^ = 0.78). There was a small effect of handgrip position for the knee, with the drops leading to decreased knee joint work compared to the tops (p<0.01, η_g_^2^ = 0.03). There was also an interaction effect between athlete level and external work rate on hip joint work (p<0.01, η_g_^2^ = 0.45) with the professional cyclists having a greater increase in hip joint work at WR_lt_ compared to 200W. For the knee, there was also a small interaction effect between handgrip position and external work rate (p<0.01, η_g_^2^ = 0.05), where the drops led to less knee joint work compared to the tops at WR_lt_. The interaction effect between handgrip and external work rate was also seen for the ankle work but the effect size was trivial (p<0.01, η_g_^2^ = 0.009).

**Table 2:** Joint work per pedal revolution for the hip, knee and ankle joints of the right leg for the recreational and professional cyclists.

Recreational		100 W			200 W			WR_lt_			*g*_*s*_
	Tops	6.8	±	5.1	23.4	±	9.8*	35.1	±	10.7*#	
Hip	Hoods	6.5	±	4.9	23.3	±	10.0*	36.8	±	11.1*#	
	Drops	6.3	±	4.6	24.2	±	9.0*	35.2	±	10.6*#	
	Tops	25.9	±	3.7	36.9	±	4.3*	44.4	±	5.1*	
Knee	Hoods	25.7	±	4.1	36.9	±	4.8*	41.8	±	5.5*‡	*0*.*44*
	Drops	25.5	±	3.6	35.2	±	4.2*	40.9	±	4.9*‡	*0*.*63*
	Tops	3.0	±	2.3	6.6	±	3.1*	9.9	±	3.8*	
Ankle	Hoods	2.8	±	2.2	6.4	±	3.2*	9.2	±	3.5*	
	Drops	2.9	±	2.1	6.3	±	3.2*	9.1	±	3.6*‡	0.12
Professional		100 W			200 W			WR_lt_			*g*_*s*_
	Tops	4.3	±	3.4	21.1	±	6.2*	51.4	±	6.2*#	
Hip	Hoods	3.8	±	3.5	19.9	±	7.8*	50.7	±	7.8*#	
	Drops	3.4	±	3.4	19.7	±	7.6*	50.7	±	8.6*#	
	Tops	26.3	±	4.5	35.4	±	4.4*	49.0	±	9.6*	
Knee	Hoods	26.8	±	4.5	35.9	±	4.7*	47.0	±	7.9*‡	0.21
	Drops	27.2	±	4.3	35.7	±	4.8*	45.2	±	7.8*‡	0.39
	Tops	2.5	±	2.3	5.2	±	2.7*	10.0	±	3.8*	
Ankle	Hoods	2.7	±	2.1	5.3	±	2.8*	10.0	±	3.4*	
	Drops	2.9	±	2.2	5.6	±	2.7*	9.6	±	3.5*‡	0.15

Mean joint work ± standard deviation (in joules). WR_lt_ = external work rate corresponding to a blood lactate of 4mmol/l.

* = significantly different from previous external work rate (p<0.05).

^#^ = indicates a significant interaction effect between external work rate and athlete level.

^‡^ = significantly different from tops (p<0.05). g_s_ = Hedges g_s_ corresponding to the pairwise comparisons between handgrip positions.

### 3.4 Crank torque

**Fig 3:**
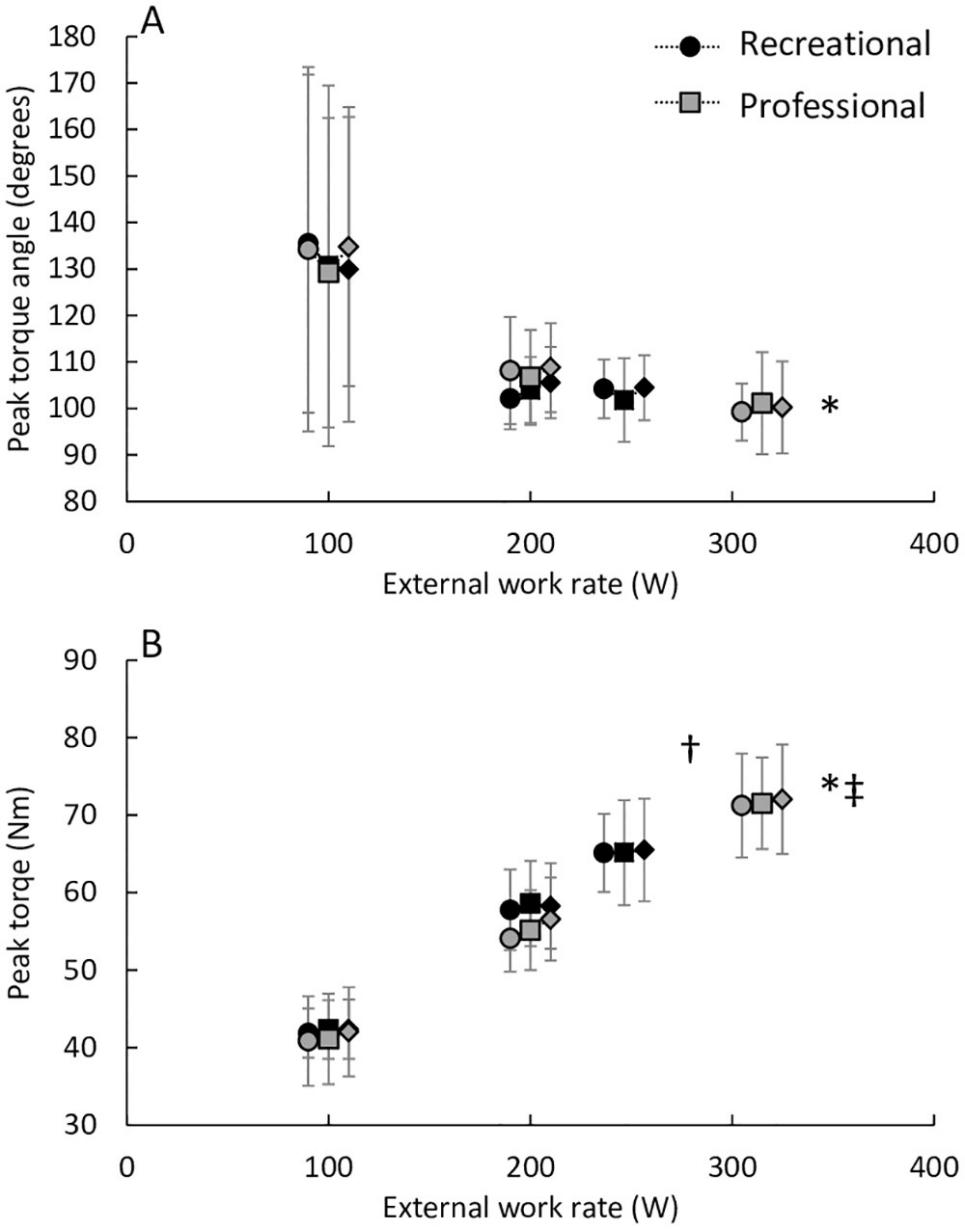
A: Angle of peak crank torque and B: peak crank torque plotted at a function of absolute external work rate. The different handgrip position are denoted by circles (tops), squares (hoods) and diamonds (drops). * indicates a significant main effect of external work rate, ‡ indicates a significant main effect of changing handgrip position, ^#^ indicates a significant main effect of performance level and † indicates a significant interaction effect between external work rate and performance level.

Both handgrip position (p < 0.05, η_g_^2^ = 0.02) and external work rate (p < 0.01, η_g_^2^ = 0.92) had a small and large effect respectively on peak crank torque (Fig 3). There was also an interaction effect of athlete level and external work rate (p < 0.01, η_g_^2^ = 0.29). Pairwise comparisons showed that when comparing 200W to WR_lt_, the professional cyclists had a greater increase in peak torque compared to the recreational cyclists (Mean increase 16.3 ± 3.8 vs 7.1 ± 4.0 Nm respectively, t = 5.03, p < 0.01, g_s_ = 2.26). Increased external work rate decreased peak crank torque angle (Fig 3: p < 0.01, η_g_^2^ = 0.44) i.e. peak crank torque occurred earlier during the down stroke but there was no effect of handgrip position (p = 0.53, η_g_^2^ < 0.01).

### 3.5 Kinematics

**Fig 4:**
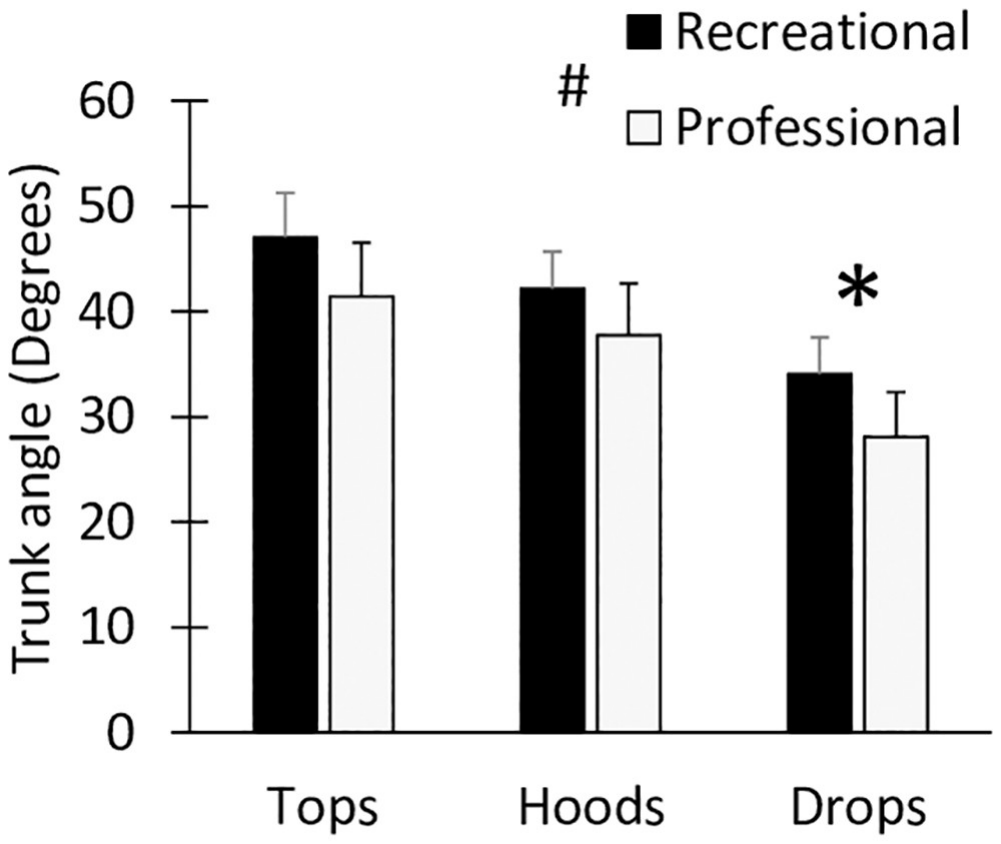
Mean back angle (i.e. absolute angle between the hip, neck and horizontal) in the tops, hoods and drops position for the recreational (black) and professional (grey) cyclists. * indicates a significant difference from previous handgrip position and ^#^ indicates a significant effect of performance level.

There was a large main effect of changing handgrip position from the tops to the hoods and drops leading to a reduced trunk angle (Fig 4: p < 0.01, η_g_^2^ = 0.89). There was also a moderate main effect of athlete level (p < 0.05, η_g_^2^ = 0.17) and the recreational riders had an average of 5.4 degrees steeper trunk angle (i.e. they were sitting more upright) compared to the professional riders at the same handgrip position.

**Fig 5:**
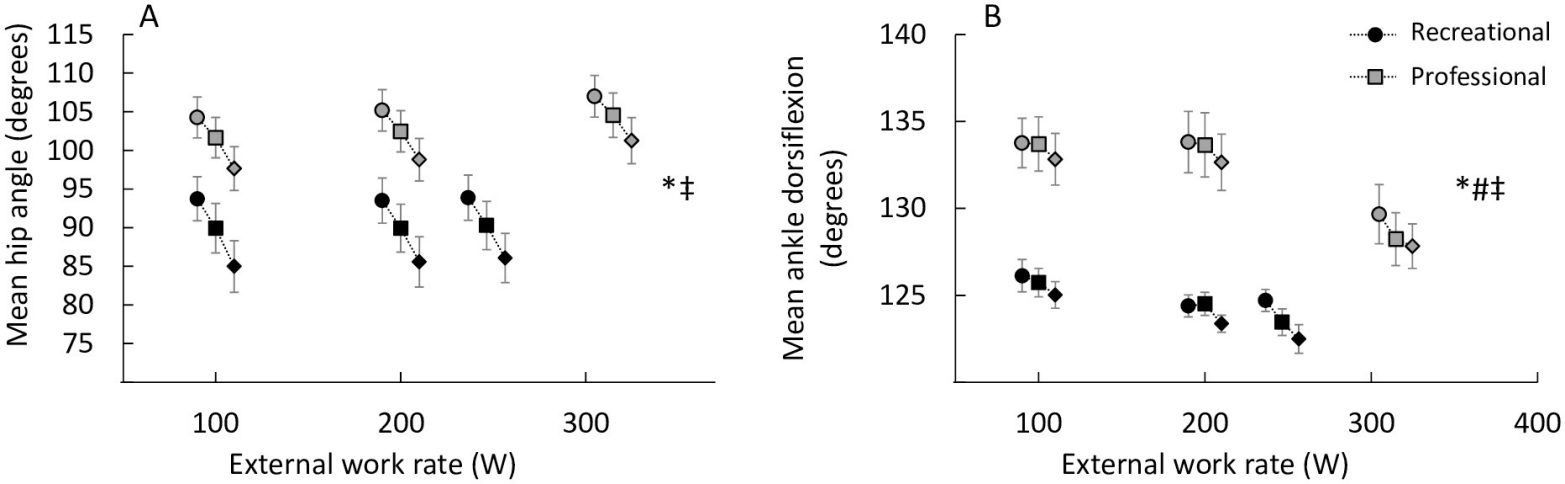
A: Mean hip angle (calculated as the angle between the pelvic, hip and knee) and B: Mean ankle angle (calculated as the angle between the knee, ankle and pedal spindle) in the different handgrip position, denoted by circles (tops), squares (hoods) and diamonds (drops) for the recreational (grey) and professional (black) cyclists. * indicates a significant main effect of external work rate, ‡ indicates a significant effect of changing handgrip position, ^#^ indicates a significant effect of performance level and † indicates a significant interaction effect between external work rate and handgrip position.

There was a small main effect of changing handgrip position (p < 0.01, η_g_^2^ = 0.05) and external work rate (p < 0.01, η_g_^2^ = 0.26) on mean ankle joint angle (Fig 5) with the drops leading to an increased mean dorsiflexion compared to the tops. There was also a large main effect of athlete level (p < 0.01, η_g_^2^ = 0.20) and the recreational riders had 7.4 degrees of increased dorsiflexion in the ankle compared to the professional riders at the same handgrip position.

There was a large main effect of changing handgrip position (p < 0.01, η_g_^2^ = 0.31) leading to decreased mean hip angle (i.e. more hip flexion) and a small effect of external work rate (p < 0.01, η_g_^2^ = 0.03) leading to increased mean hip angle (Fig 5). There was no significant effect of athlete level (p = 0.06, η_g_^2^ = 0.19).

There was a small main effect of changing handgrip position on hip ROM (p < 0.05, η_g_^2^ = 0.03) with the drops leading to a 0.4 degrees smaller ROM compared to tops and external work rate (p < 0.05, η_g_^2^ = 0.10) with WR_lt_ leading to 0.8 degrees smaller ROM compared to 100W. There was a large effect of athlete level (p < 0.01, η_g_^2^ = 0.29), where the professional cyclists had a 3.9 degrees larger ROM at the same handgrip position.

There was also a large main effect of changing external work rate (p < 0.01, η_g_^2^ = 0.20) on ankle ROM with WRlt led to 4.0 degrees greater ROM compared to 100W, but there was no significant effect of athlete level (p = 0.98, η_g_^2^ < 0.01) or handgrip position (p = 0.80, η_g_^2^ < 0.001).

## 4. Discussion

The aim of this study was to investigate the effects of using different handgrip positions, at different external work rates during seated cycling on the relative joint specific power contribution in cyclists of different performance levels. The main finding was that changing handgrip position had no main effect on joint power in any joint. Independent of handgrip position, there were large effects of changing external work rate on relative hip and knee joint specific power and the different performance levels due to differences in power output at LT. However, there was a small effect of changing handgrip position when comparing across large ranges in external work rate (i.e. 100W vs. WR_lt_). Additionally, changing handgrip position from the tops to the drops led to a small increase in mean dorsiflexion angle, a moderate increase in mean hip flexion angle and a small increase in peak crank torque.

When interpreting these findings, it is important to consider pedal forces, joint powers and kinematic changes as connected. Using the drops reduced the mean hip angle but an increased dorsal flexion (i.e. heel drop) counteracted the effect by allowing for the increased hip extension relative to horizontal and thus the increased dorsiflexion may function to maintain hip ROM. The maintained ROM may allow the primary work producing hip muscles to sustain normal function and thus limit the changes in joint specific contribution resulting from changing position from the tops to the drops. Although the heel drop may not be able to completely counteract the reduced hip angle and the effect on hip joint power contribution that occurs when using the drops, it might be enough to lead to the limited influence demonstrated by the small effect seen on joint specific power.

Comparing our results with those of Bini et al. [15] obtained during sprint cycling using different handlebar heights, we found smaller effect sizes and we only found effects on relative hip contribution with a decreased hip contribution at 100W, and increased hip contribution at WR_lt_ while Bini et al. [15] found no changes in hip or ankle mean angles. It is possible that the changes we found in ankle and hip mean angle may only be effective at maintaining joint power at submaximal intensities and positions such as the tops and drops and not during more extreme sprinting positions and external work rates.

We achieved the desired reduction in back and hip angle by using the drops handgrip position and although the recreational cyclists had [21] a steeper trunk angle compared to the professional cyclists, the effect of changing handgrip position was similar in both groups. The reduced trunk angle seen in the professional riders may be due to their increased participation in races, where a low trunk is often preferred for reducing the effect of wind resistance [21].

Changing handgrip position from the tops to the drops led to an increase in peak crank torque for both groups, but no change in the timing of peak crank torque. Our finding of increased peak crank torque when using the drop position is in accordance with previous literature [8]. However, previous studies have also found delayed peak crank torque in the pedal cycle [6, 8]. Our study differs from these mentioned studies in that we did not use a time trial specific position and indeed Dorel et al. [6] did not report an effect on peak crank torque angle of using the drop position similar to the one used in this study. A time trial specific position would elicit a greater change in position from the tops and could potentially explain this discrepancy in findings between our study and the literature.

We found no differences between performance levels in the effect of using the drops compared to the hoods and tops on joint specific power. The small effect of handgrip position on joint specific power does not seem to explain the physiological changes reported previously resulting from utilizing the drops or a time trial specific position [3, 4]. It is possible that there is a higher metabolic cost although the cyclists are able to produce the same joint power and whole body energetic measurements in combination with local muscle measurements of blood flow or muscle oxygenation could provide some additional insight on the metabolic aspects. Additionally, a longer duration trial could potentially elucidate if the handgrip positions influence joint power when fatigue accumulates. However, we compared recreational cyclists to professional cyclists and although there are clear differences in the power output they are able to produce at WR_lt_, they are all familiar with cycling and using the different position of a road bike handlebar. It is possible that only a minor amount of cycling experience in necessary for achieving a joint specific power distribution robust to further improvements in cycling experience. This would warrant the investigation of the effect of trunk angle when cycling in a population not familiar with the different positions caused by a road bike handlebar.

The professional cyclists had a greater hip joint contribution at WR_lt_ compared to the recreational cyclists. An increased reliance on hip joint contribution at higher external work rate has been previously shown [9] and thus, the effect is likely due to the increased external work rate used by the professionals in the WR_lt_ condition. In accordance, peak crank torque increased when external work rate increased and there were no differences between the two groups with the exception of a greater increase in the professional compared to the recreational at the LT compared to the 200 W conditions. When considering the WR_lt_ is greater for the professional group compared to the recreational group this finding is not surprising.

Taken together, there were no differences between the professional and recreational riders in peak torque or the timing of peak torque at 100W and 200W but only WR_lt_. However, as with joint specific power, the differences occurred largely as an effect of increased external work rate used by the professionals at WR_lt_. These finding shows that at the same absolute power output, recreational and professional cyclists will have a similar joint specific power contribution at least in the low to moderate external range used in the present study. However, at an individualized external power output such as WR_lt_ the joint power contribution will differ due to the different absolute power output and not due to the differences in performance levels. Although the recreational riders in this study were at a much lower performance level than the professional riders, they were still well familiar with cycling and do have a much larger training volume compared to an untrained participants who also may be unfamiliar with the pedalling exercise. The findings on pedal peak torque, the timing of peak torque and relative joint power also indicates that only a certain amount of cycling experience is necessary to generate a pedalling pattern which changes little with subsequent performance increases at least when investigated at the same absolute power output.

The use of a freely chosen cadence and a minimum of instructions for the cyclists presents both challenges and advantages. The major advantage of this approach is that the cyclists were able to maintain their own pedalling style and cadence. To the best of our knowledge, the effect of a locked cadence compared to a freely chosen cadence on joint specific power has yet to be investigated. A large change in cadence could have led to changes in joint power [12]. The reduction in cadence (i.e. 6.4 RPM) between different power outputs could have influenced joint power but this reduction should have increased relative hip joint contribution and thus may overestimate the effect of the present study. However, the difference in the change of cadence between the recreational and professional cyclists was 4.0 rpm and based on previous work by our group [12] this would result in a hip and knee joint power change of ~3W and approximately half of the change elicited by a change of 25W external power [9]. Furthermore, the cadence freely chosen by the recreational cyclists was slightly lower compared to the professional (i.e. 90.3 vs 91.8). Although a small difference, this could possibly lead to a small underestimation of the difference between the groups at WR_lt_ [13].

Since we used an ergometer providing constant power, a decreased cadence would require more work per cycle to achieve the same external work rate and thus presenting joint work provides a more complete picture of the effect. Beyond the effect of external work rate leading to increased joint work, there was a moderate decrease in knee joint work and a small decrease ankle joint work at WR_lt_ when changing from the tops to the hoods. The findings on joint work demonstrate that the increased hip joint contribution results from maintained hip joint work and reduced knee and ankle joint work. The reduced total work would be partially explained by the 1.6 RPM cadence increase, but potentially also by increased upper body or lateral leg movements not fully detected by the current 2D inverse dynamics model.

Our approach of simply instructing the participants to change handgrip position ensures that the most “natural” cycling pattern is maintained but opens the possibility for differences in how the participants changed handgrip position, (e.g. scapular movement instead of back and hip movement in order to extend reach). However, by only instructing a changing handgrip position, we achieved similar changes in trunk angle in both groups (i.e. a reduction in trunk angle by 8.1±3.0 and 9.5±2.3 degrees for the professional and recreational groups respectively) and no changes between different power outputs. The changes observed in hip angle is comparable with previous reports in the literature [5].

Some additional limitations include the use of 2D force inputs and inverse dynamics which may not fully capture the 3D movements of the joints. Furthermore, the reflective markers are placed on the skin and does not exactly reflect the rotational points of the joints in the joint centres. Finally, it should be mentioned that it is problematic to recruit professional cyclists for research projects and thus the sample size in low and we could not follow traditional sample size calculation.

In conclusion, this study demonstrates no main effect of changing handgrip position from the tops to the drops in professional and recreational cyclists. However, there seems to be a small effect on hip joint power when comparing across large ranges in external work rates. Changing handgrip position has a small effect on peak crank torque and an on ankle mean angles as well as a moderate effect on hip mean angle. Although we cannot directly conclude on implications for performance, most of the effects on hip joint power and joint kinematics are small or moderate and any negative effect on performance that may exist is likely outweighed by the aerodynamic benefit of using the drops.

## Supporting information

S1 Data (XLSX)
